# Supplementary material for: Diagnostic and prognostic factors in patients with prostate cancer: a systematic review protocol
Source: BMJ Open. 2021 Feb 11;11(2):e040531. doi: 10.1136/bmjopen-2020-040531 (PMC7880102; doi:10.1136/bmjopen-2020-040531)
Supplement: Author's manuscript [file bmjopen-2020-040531.draft_revisions.pdf]

BMJ Open is committed to open peer review. As part of this commitment we make the peer review history of every article we publish publicly available.

When an article is published we post the peer reviewers' comments and the authors' responses online. We also post the versions of the paper that were used during peer review. These are the versions that the peer review comments apply to.

The versions of the paper that follow are the versions that were submitted during the peer review process. They are not the versions of record or the final published versions. They should not be cited or distributed as the published version of this manuscript.

BMJ Open is an open access journal and the full, final, typeset and author-corrected version of record of the manuscript is available on our site with no access controls, subscription charges or pay-per-view fees (http://bmjopen.bmj.com).

If you have any questions on BMJ Open's open peer review process please email info.bmjopen@bmj.com

BMJ Open

A systematic review protocol of diagnostic and prognostic factors in patients with prostate cancer

Journal:	*BMJ Open*
Manuscript ID	bmjopen-2020-040531
Article Type:	Protocol
Date Submitted by the Author:	19-May-2020
Complete List of Authors:	Beyer, Katharina; King's College London, Translational Oncology and Urology Research (TOUR)<br>Moris, Lisa; KU Leuven University Hospitals Leuven, Department of Urology<br>Lardas, Michael; Metropolitan Hospital Athens, Department of Urology<br>Haire, Anna ; King's College London, Translational Oncology and Urology Research (TOUR)<br>Barletta, Francesco; IRCCS Ospedale San Raffaele, Unit of Urology/Division of Oncology, URI<br>Scuderi, Simone; IRCCS Ospedale San Raffaele, Unit of Urology/Division of Oncology, URI<br>Vradi, Eleni; Bayer Pharma AG, Medical Affairs and PV<br>Gandaglia, Giorgio; IRCCS Ospedale San Raffaele, Unit of Urology/Division of Oncology, URI<br>Omar, Muhammad; University of Aberdeen, Academic Urology Unit, Health Services Research Unit<br>MacLennan, Steven; University of Aberdeen, Academic Urology Unit, Health Services Research Unit<br>Zong, Jihong; Bayer U.S. , Epidemiology<br>Farahmand, Bahman; Bayer AG<br>Maclennan, Sara; University of Aberdeen, Academic Urology Unit, Health Services Research Unit<br>Devecseri, Zsuzsanna; Sanofi SA<br>Asiimwe, Alex; Bayer Pharma AG, Medical Affairs and PV<br>Collette, Laurence ; EORTC<br>Bjartell, Anders; Skåne University Hospital,<br>NDow, James; University of Aberdeen, Surgery<br>Briganti , A; Vita-Salute University San Raffaele; IRCCS Ospedale San Raffaele, Unit of Urology/Division of Oncology, URI<br>Van Hemelrijck, Mieke; King's College London, Translational Oncology and Urology Research (TOUR)
Keywords:	Prostate disease < UROLOGY, ONCOLOGY, Urological tumours < UROLOGY

I, the Submitting Author has the right to grant and does grant on behalf of all authors of the Work (as defined in the below author licence), an exclusive licence and/or a non-exclusive licence for contributions from authors who are: i) UK Crown employees; ii) where BMJ has agreed a CC-BY licence shall apply, and/or iii) in accordance with the terms applicable for US Federal Government officers or employees acting as part of their official duties; on a worldwide, perpetual, irrevocable, royalty-free basis to BMJ Publishing Group Ltd ("BMJ") its licensees and where the relevant Journal is co-owned by BMJ to the co-owners of the Journal, to publish the Work in this journal and any other BMJ products and to exploit all rights, as set out in our licence.

The Submitting Author accepts and understands that any supply made under these terms is made by BMJ to the Submitting Author unless you are acting as an employee on behalf of your employer or a postgraduate student of an affiliated institution which is paying any applicable article publishing charge ("APC") for Open Access articles. Where the Submitting Author wishes to make the Work available on an Open Access basis (and intends to pay the relevant APC), the terms of reuse of such Open Access shall be governed by a Creative Commons licence – details of these licences and which Creative Commons licence will apply to this Work are set out in our licence referred to above.

Other than as permitted in any relevant BMJ Author's Self Archiving Policies, I confirm this Work has not been accepted for publication elsewhere, is not being considered for publication elsewhere and does not duplicate material already published. I confirm all authors consent to publication of this Work and authorise the granting of this licence.

For peer review only

A systematic review protocol of diagnostic and prognostic factors in patients with prostate cancer

Author list: Katharina Beyer[a]; Lisa Moris[b]; Michael Lardas[c]; Anna Haire[a]; Francesco Barletta[d] ; Simone Scuderi[d]; Eleni Vradi[e]; Giorgio Gandaglia[d]; Muhammad Imran Omar[f]; Steven Maclennan[f]; Jihong Zong[g]; Bahman Farahmand[h]; Sara J Maclennan[f]; Zsuzsanna Devecseri[i], Alex Asiimwe[e]; Laurence Collette[j]; Anders Bjartell[k], James N'Dow[f]; Alberto Briganti[d,l]; Mieke Van Hemelrijck[a] and PIONEER Consortium*

Corresponding author:

Katharina Beyer,
Translational Oncology and Urology Research (TOUR),
School of Cancer and Pharmaceutical Studies,
3rd Floor, Bermondsey Wing, Guy's Hospital, Great Maze Pond,
London SE1 9RT,
02071885594
katharina.beyer@kcl.ac.uk

Affiliations:

[a] Translational and Oncology Research (TOUR), Faculty of Life Sciences and Medicine, King's College London, London, UK
[b] Department of Urology, University Hospitals Leuven, Leuven, Belgium
[c] Department of Urology, Metropolitan general, Athens, Greece
[d] Unit of Urology/Division of Oncology, URI, IRCCS Ospedale San Raffaele, Milan, Italy
[e] Department of Epidemiology, Bayer AG, Berlin, Germany
[f] Academic Urology Unit, Health Services Research Unit, University of Aberdeen, Aberdeen, UK
[g] Global Epidemiology, Bayer HealthCare Pharmaceuticals Inc., Whippany, USA
[h] Global Epidemiology, Bayer AB, Stockholm, Sweden
[i] Sanofi, Paris, France
[j] EORTC Headquarters, Brussels, Belgium
[k] Department of Translational Medicine, Medical Faculty, Lund University, Lund University, Malmo, Sweden
[l] Department of Urology, University Vita e Salute-San Raffaele, Milan, Italy

Key words: Prostate cancer, diagnostic factor, prognostic factors
Word count of text: 1629

For peer review only

1. Introduction

Prostate cancer (PCa) is the second most common cancer worldwide [1] and accounts for 15% of all cancers diagnosed [2]. Clinically localised PCa is typically characterised by a favourable long-term natural history, where several therapeutic options are available. Moreover, the treatment landscape of advanced and metastatic PCa has changed dramatically in the past decade with the approval of multiple systemic agents, improving patients' survival. PCa is usually suspected based upon the clinical findings of digital rectal examination (DRE) and/or Prostate Specific Antigen (PSA) level [2]. However, which treatment strategy is best or which biomarkers can be used to select patients for specific therapeutic options remains largely uncertain [3]. Multiple diagnostic and prognostic factors (DPFs) are available on top of traditional PSA testing to improve the diagnosis of PCa, such as PCA3, TMPRSS2-ERG fusion, or kallikreins as incorporated in the Phi or 4Kscore test [4-7]. However, the European Association of Urology (EAU) guidelines (2019) currently do not provide recommendations to implement these factors or biomarkers into routine screening programmes due to limited data [2].

The PIONEER Consortium is an international collaboration coordinated by the EAU which aims to establish the best evidence-based management and clinical practice of PCa across all disease stages using the power of big data analytics towards a more outcome-driven, value-based, and patient-centric healthcare system. A key objective is to address one of the major challenges within the context of diagnostic or prognostic biomarkers/factors: the inability to incorporate real-world clinical outcome data into the management of PCa in terms of screening, diagnosis and treatment. Biomarkers can be classified into different types: diagnostic, prognostic, predictive, and therapeutic. A diagnostic biomarker or factor allows the early detection of cancer in a non-invasive way and thus the secondary prevention of cancer [8]. A prognostic biomarker or factor is a clinical or biological characteristic that provides information on the likely course of the disease [8]. In the sections below we have used the terms biomarkers and factors interchangeably.

This project aims to investigate which DPFs are available in relation to previously defined outcomes for PCa [9-13] by looking at the evidence published from 2014 onwards to reflect

For peer review only - http://bmjopen.bmj.com/site/about/guidelines.xhtml

current medical practice and the '2014 International Society of Urological Pathology (ISUP) Consensus Conference on Grading Patterns and Proposal for a New Grading system'[14].

2. Methods and analysis

This research project will follow the following four steps (Figure 1):

(1) We will start with a broad systematic literature-based review of DPFs for all stages of PCa, covering English language publications of human studies from 2014 onwards.

(2) The final list of all available DPFs for which a systematic review is required will then be discussed by a multidisciplinary expert panel.

(3) Each study and systematic review identified through the literature search will be assessed using a risk of bias tool and if applicable the PROBAST (Prediction model Risk Of Bias ASsessment Tool) criteria.

(4) For those studies with an overall low score on risk of bias and low concerns for the applicability of their results, we will use the Classification from the Oxford Centre for Evidence Based Medicine to define whether an additional quantitative assessment is required.

2.1. Stage 1: Systematic review

The systematic review will be conducted in accordance with the Preferred Reporting Items for Systematic Reviews and MetaAnalyses (PRISMA) guidelines [15 16].

2.1.1. Search methods and identification of studies:

The literature search has been developed by an experienced Information Scientist. We will search for quantitative observational studies which assess either diagnostic or prognostic factors. The search strategy is shown in Table 1.

The eligibility will be independently checked by at least two researchers. Conflicts will be solved by discussion or consulting a third party. The abstract and full-text screening will be conducted in duplicate, and results will be compared and shared with the core group involved in the research project. The EAU guidelines will also be hand-searched to identify DPFs [2].

For peer review only - http://bmjopen.bmj.com/site/about/guidelines.xhtml

2.1.2. Eligibility criteria

Types of studies: We will include quantitative studies only. Studies included in this systematic review will be limited to those which are published between January 2014 and January 2020, so that the project is pragmatic. Qualitative studies, narrative literature reviews and commentary pieces will be excluded. Single studies with fewer than 50 participants will be excluded since a small patient number is unlikely to influence practice due to lack of power and risk of selection bias in the study. In addition, the test is unlikely to be ready for clinical usage. Studies which are not published in English language or are out of our defined timeframe will also be excluded. We believe that DPFs which are relevant to clinical practice and are developed outside of the proposed timeframe will either be captured in the EAU guidelines [2] or are already included in the evidence reviewed due to high relevance.

Types of participants: The participants are adult men (≥18 years of age) diagnosed with:

a) Localised PCa: ≤T2cN0M0 with no treatment prior to their primary treatment for PCa (except for neoadjuvant androgen deprivation therapy [ADT] preceding radiotherapy). Studies in which locally advanced or metastatic disease (T3-T4 N+ or M+) accounted for >10% of their participants will be excluded unless the localised participants are reported separately.
b) Locally advanced PCa: T3 or T4 and/or N1.
c) Metastatic PCa: any T, any N, M1 a-c. Studies in which local disease (≤ any T, any N, M0) accounted for >10% of their participants will be excluded, unless the metastatic participants are reported separately.
d) nmCRPC: any T, N, M0 and castration-resistance defined as a castrate serum testosterone < 50 ng/dL or 1.7 nmol/L plus either biochemical or radiological progression.

Types of interventions: Interventions considered for this systematic review will all be interventions supported by the 2019 EAU guidelines [2] for localised, locally advanced, metastatic and non-metastatic castration resistant PCa.

For peer review only - http://bmjopen.bmj.com/site/about/guidelines.xhtml

Data extraction: Data will be extracted by at least two reviewers and checked by a third reviewer following the Critical Appraisal and Data Extraction for systematic reviews of prediction modelling studies (CHARMS PF Checklist)[17 18].

2.2. Stage 2: Assessment of Stage 1 output with expert panel to identify the individual topics for systematic reviews.

An expert panel of urologists, radiologists, radiation oncologist, oncologist, methodologist and pathologists will review the extracted factors to discuss if any DPFs are missing. If no DPFs are missing, the review team will assess the quality of the identified studies for each DPF systematic review using the PROBAST (Prediction model Risk Of Bias ASsessment Tool) criteria [19 20].

2.3. Stage 3: Risk of Bias assessment of individual articles using PROBAST

Each study and systematic review identified through the literature search will be assessed using PROBAST (Prediction model Risk Of Bias ASsessment Tool) criteria [19]. PROBAST is a tool to assess the risk of bias as well as the applicability of diagnostic and prognostic prediction models. For studies that will not meet the PROBAST criteria, we will use QUADAS-2 [21] for diagnostic factors accuracy studies and QUIPS[22] for prognostic factors studies.

2.4. Stage 4: Quantitative assessment of individual articles using meta-analytic techniques

We are well aware of the limitation of pooling evidence from non-randomised studies, as there could be specific bias inherent within the design. We will be very cautious while pooling evidence from non-randomised studies. For those studies with an overall low score on risk of bias and low on concerns for applicability, we will use the Classification from the Oxford Centre for Evidence Based Medicine to define whether an additional quantitative assessment is required.

1. If there is Level 1a (SR of inception cohort studies validated in different populations or Level 1 diagnostic studies from different clinical centres), we do not do a meta-analysis.

For peer review only - http://bmjopen.bmj.com/site/about/guidelines.xhtml

2. No Level 1a but >2 studies which apply to 1b (Prognostic: individual inception cohort study with > 80% follow-up; Clinical Decision Rule (CDR) validated in a single population; Diagnostic: Validating cohort study with good reference standards; or CDR tested within one clinical centre) or 1c (Prognostic: All or none case-series; Diagnostic: Absolute specificity is so high that a positive result rules-in the diagnosis (SpPins) and diagnostic finding whose Sensitivity is so high that a Negative result rules-out the diagnosis (SnNouts)), we will perform a meta-analysis.

3. Ethics and Dissemination

No additional ethical approval is required as the work will rely on published publicly available study data.

The findings of all these systematic reviews will be exported into an online search tool to ensure wide applicability of the study findings. More specifically, this online search tool will produce evidence-based recommendations so that these could be used by researchers, clinicians and experts in the field. The tool will be designed so that stakeholders can access up to date available evidence (and view the quality of the studies published) when developing new DPFs or setting up clinical trials. To ensure sustainability of this tool, PIONEER and the EAU aim to update the systematic reviews described above on a regular basis to reflect the latest available research on DPFs for PCa.

For peer review only - http://bmjopen.bmj.com/site/about/guidelines.xhtml

For peer review only

References

1. Bray F, Ferlay J, Soerjomataram I, et al. Global cancer statistics 2018: GLOBOCAN estimates of incidence and mortality worldwide for 36 cancers in 185 countries. *CA: a cancer journal for clinicians* 2018;68(6):394-424. doi: 10.3322/caac.21492 [published Online First: 2018/09/13]
2. Mottet N, Bellmunt J, Bolla M, et al. EAU-ESTRO-SIOG Guidelines on Prostate Cancer. Part 1: Screening, Diagnosis, and Local Treatment with Curative Intent. *European urology* 2017;71(4):618-29. doi: 10.1016/j.eururo.2016.08.003 [published Online First: 2016/08/30]
3. Filella X, Fernandez-Galan E, Fernandez Bonifacio R, et al. Emerging biomarkers in the diagnosis of prostate cancer. *Pharmacogenomics and personalized medicine* 2018;11:83-94. doi: 10.2147/pgpm.S136026 [published Online First: 2018/05/31]
4. Vedder MM, de Bekker-Grob EW, Lilja HG, et al. The added value of percentage of free to total prostate-specific antigen, PCA3, and a kallikrein panel to the ERSPC risk calculator for prostate cancer in prescreened men. *European urology* 2014;66(6):1109-15. doi: 10.1016/j.eururo.2014.08.011 [published Online First: 2014/08/30]
5. Leyten GH, Hessels D, Jannink SA, et al. Prospective multicentre evaluation of PCA3 and TMPRSS2-ERG gene fusions as diagnostic and prognostic urinary biomarkers for prostate cancer. *European urology* 2014;65(3):534-42. doi: 10.1016/j.eururo.2012.11.014 [published Online First: 2012/12/04]
6. Boegemann M, Stephan C, Cammann H, et al. The percentage of prostate-specific antigen (PSA) isoform [-2]proPSA and the Prostate Health Index improve the diagnostic accuracy for clinically relevant prostate cancer at initial and repeat biopsy compared with total PSA and percentage free PSA in men aged </=65 years. *BJU international* 2016;117(1):72-9. doi: 10.1111/bju.13139 [published Online First: 2015/03/31]
7. Bryant RJ, Sjoberg DD, Vickers AJ, et al. Predicting high-grade cancer at ten-core prostate biopsy using four kallikrein markers measured in blood in the ProtecT study. *Journal of the National Cancer Institute* 2015;107(7) doi: 10.1093/jnci/djv095 [published Online First: 2015/04/12]
8. Carlomagno N, Incollingo P, Tammaro V, et al. Diagnostic, Predictive, Prognostic, and Therapeutic Molecular Biomarkers in Third Millennium: A Breakthrough in Gastric Cancer. *BioMed research international* 2017;2017:7869802. doi: 10.1155/2017/7869802 [published Online First: 2017/11/03]
9. Beyer K ML, Lardas M, Maclennan S, Monagas S, Antunes-Lopes T, et al. PIONEER's systematic review of outcomes reported in effectiveness trials for interventions in locally advanced prostate cancer. 11th European Multidisciplinary Congress on Urological Cancers. 11th European Multidisciplinary Congress on Urological Cancers. Vienna, 2019.
10. Beyer K MS, Lardas M, Moris L, Omar M, Flaherty S. PIONEER's update and integration of a localised prostate cancer core outcome set for effectiveness trials and a standard set for clinical practice. Annual Meeting of the European Association of Urology. Annual Meeting of the European Association of Urology. Barcelona2019, 2019.
11. Beyer K MS, Lardas M, Moris L, Vradi E, Picozzi M, et al. . PIONEER's update and integration of a metastatic Prostate Cancer Core Outcome Set. ICHOM 2019. Rotterdam, 2019.
12. Beyer K MS, Lardas M, Moris L, Omar M, MacLennan S, et al. PIONEER's update and integration of a localised prostate cancer core outcome set for effectiveness trials and a standard set for clinical practice. 11th European Multidisciplinary Congress on Urological Cancers. 11th European Multidisciplinary Congress on Urological Cancers. Vienna, 2019.
13. Beyer K MS, Lardas M, Moris L, Vradi E, Omar, I, et al PIONEER's systematic review of outcomes in RCTs of men with non-metastatic castration resistant prostate cancer. Is there a need for a core outcome set? Annual Meeting of the European Association of Urology. Amsterdam, 2020.
14. Epstein JI, Egevad L, Amin MB, et al. The 2014 International Society of Urological Pathology (ISUP) Consensus Conference on Gleason Grading of Prostatic Carcinoma: Definition of Grading Patterns and Proposal for a New Grading System. *The American journal of surgical*

For peer review only - http://bmjopen.bmj.com/site/about/guidelines.xhtml

pathology 2016;40(2):244-52. doi: 10.1097/pas.0000000000000530 [published Online First: 2015/10/23]
15. Moher D, Liberati A, Tetzlaff J, et al. Preferred reporting items for systematic reviews and meta-analyses: the PRISMA statement. *PLoS medicine* 2009;6(7):e1000097-e97. doi: 10.1371/journal.pmed.1000097
16. Higgins JPT TJ, Chandler J, Cumpston M, Li T, Page MJ, Welch VA Cochrane Handbook for Systematic Reviews of Interventions. Chichester (UK): Cochrane, 2019.
17. Moons KGM, de Groot JAH, Bouwmeester W, et al. Critical Appraisal and Data Extraction for Systematic Reviews of Prediction Modelling Studies: The CHARMS Checklist. *PLOS Medicine* 2014;11(10):e1001744. doi: 10.1371/journal.pmed.1001744
18. Riley RD, Moons KGM, Snell KIE, et al. A guide to systematic review and meta-analysis of prognostic factor studies. *BMJ (Clinical research ed)* 2019;364:k4597. doi: 10.1136/bmj.k4597 [published Online First: 2019/02/01]
19. Wolff RF, Moons KGM, Riley RD, et al. PROBAST: A Tool to Assess the Risk of Bias and Applicability of Prediction Model Studies. *Annals of Internal Medicine* 2019;170(1):51-58. doi: 10.7326/M18-1376
20. Moons KGM, Wolff RF, Riley RD, et al. PROBAST: A Tool to Assess Risk of Bias and Applicability of Prediction Model Studies: Explanation and Elaboration. *Ann Intern Med* 2019;170(1):W1-w33. doi: 10.7326/m18-1377 [published Online First: 2019/01/01]
21. Whiting PF, Rutjes AW, Westwood ME, et al. QUADAS-2: a revised tool for the quality assessment of diagnostic accuracy studies. *Ann Intern Med* 2011;155(8):529-36. doi: 10.7326/0003-4819-155-8-201110180-00009 [published Online First: 2011/10/19]
22. Hayden JA, van der Windt DA, Cartwright JL, et al. Assessing bias in studies of prognostic factors. *Ann Intern Med* 2013;158(4):280-6. doi: 10.7326/0003-4819-158-4-201302190-00009 [published Online First: 2013/02/20]

For peer review only

For peer review only - http://bmjopen.bmj.com/site/about/guidelines.xhtml

Tables and Figures

Table 1: Search strategy

Database: OVID Medline Epub Ahead of Print, In-Process & Other Non-Indexed Citations, Ovid MEDLINE(R) Daily and Ovid MEDLINE(R) 1946 to Present, Embase <1974 to 2020 January 28>, EBM Reviews - Cochrane Database of Systematic Reviews <2005 to January 21, 2020>

Search Strategy:

----

1 exp *Prostatic Neoplasms/ (262435)

2 exp *prostate cancer/ (245472)

3 (prostat* adj2 (cancer* or carcinoma* or malignan* or tumor* or tumour* or neoplas* or adenocarcinoma* or adenoma*)).tw. (332251)

4 or/1-3 (366427)

5 ((diagnostic or prognos* or predict*) adj10 (biomarker or biomarkers or factor or factors)).tw,kw. (717487)

6 ((diagnostic or prognos* or predict*) adj10 (Oncotype Dx Prostate or Prolaris or Decipher or Decipher PORTOS or ProMark)).tw,kw. (458)

7 5 or 6 (717869)

8 4 and 7 (17456)

9 limit 8 to english language [Limit not valid in CDSR; records were retained] (16484)

10 limit 9 to yr="2014 -Current" (8417)

11 conference abstract.pt. or Congresses as Topic/ or Conference Review.pt. or "Journal: Conference Abstract".pt. (3815712)

12 10 not 11 (5902)

13 (exp animals/ or exp animal/ or exp nonhuman/ or exp animal experiment/ or animal model/ or animal tissue/ or non human/ or (rat or rats or mice or mouse or swine or porcine or murine or sheep or lambs or pigs or piglets or rabbit or rabbits or cat or cats or dog or dogs or cattle or bovine or monkey or monkeys or trout or marmoset$1).ti.) not (humans/ or human/ or human experiment/ or (human* or men or women or patients or subjects).tw.) (10251935)

14 12 not 13 (5882)

15 note/ or editorial/ or letter/ or Comment/ or news/ or (note or editorial or letter or Comment or news).pt. (4565255)

16 14 not 15 (5811)

17 (child/ or Pediatrics/ or Adolescent/ or Infant/ or adolescence/ or newborn/ or (baby or babies or child or children or pediatric* or paediatric* or peadiatric* or infant* or infancy or neonat* or

For peer review only

For peer review only - http://bmjopen.bmj.com/site/about/guidelines.xhtml

	newborn* or new born* or adolescen* or preschool or pre-school or toddler*).tw.) not (adult/ or aged/ or (aged or adult* or elder* or senior* or men or women).tw.) (4146377)
18	16 not 17 (5794)
19	18 use ppez,oemezd (5788)
20	10 use coch (6)
21	19 or 20 (5794)
22	remove duplicates from 21 (3140)

Figure 1: Overview of four stage process

For peer review only

Figure 1: Overview of four stage process

Workflow	Task
Stage 1.	Broad literature-based systematic review of diagnostic and prognostic factors (DPs) for all stages of prostate cancer from 2014 onwards (English only; humans).<br>• Extract data from the included studies following the CHARMS-PF guideline.
Stage 2.	Discussion of systematic review findings by a multidisciplinary expert panel<br>• Review the list of included studies
Stage 3.	Risk of Bias Assessment and applicability of individual studies using PROBAST, QUIPS and QUADAS-2
Stage 4.	Quantitative assessment of individual articles using meta-analytic techniques:<br>• If PROBAST indicates low risk of bias and low concerns for applicability:<br>Oxford Classification Centre for Evidence Based Medicine:<br>1. If there is Level 1a (SR of inception cohort studies validated in different populations or Level 1 diagnostic studies from different clinical centres), we do not do a meta-analysis<br>2. No Level 1a but >2 studies which apply to 1b (Prognostic: individual inception cohort study with > 80% follow-up; CDR" validated in a single population; Diagnostic: Validating cohort study with good reference standards; or CDR" tested within one clinical centre) or 1c (Prognostic: All or none case-series; diagnostic: Absolute SpPins and SnNouts), we do a meta-analysis
Final aim:	Develop online PIONEER Online Search Tool for DPFs

For peer review only

A systematic review protocol of diagnostic and prognostic factors in patients with prostate cancer

Journal:	*BMJ Open*
Manuscript ID	bmjopen-2020-040531.R1
Article Type:	Protocol
Date Submitted by the Author:	03-Nov-2020
Complete List of Authors:	Beyer, Katharina; King's College London, Translational Oncology and Urology Research (TOUR)<br>Moris, Lisa; KU Leuven University Hospitals Leuven, Department of Urology<br>Lardas, Michael; Metropolitan Hospital Athens, Department of Urology<br>Haire, Anna ; King's College London, Translational Oncology and Urology Research (TOUR)<br>Barletta, Francesco; IRCCS Ospedale San Raffaele, Unit of Urology/Division of Oncology, URI<br>Scuderi, Simone; IRCCS Ospedale San Raffaele, Unit of Urology/Division of Oncology, URI<br>Vradi, Eleni; Bayer Pharma AG, Medical Affairs and PV<br>Gandaglia, Giorgio; IRCCS Ospedale San Raffaele, Unit of Urology/Division of Oncology, URI<br>Omar, Muhammad; University of Aberdeen, Academic Urology Unit, Health Services Research Unit<br>MacLennan, Steven; University of Aberdeen, Academic Urology Unit, Health Services Research Unit<br>Zong, Jihong; Bayer U.S. , Epidemiology<br>Farahmand, Bahman; Bayer AG<br>Maclennan, Sara; University of Aberdeen, Academic Urology Unit, Health Services Research Unit<br>Devecseri, Zsuzsanna; Sanofi SA<br>Asiimwe, Alex; Bayer Pharma AG, Medical Affairs and PV<br>Collette, Laurence ; EORTC<br>Bjartell, Anders; Skåne University Hospital,<br>NDow, James; University of Aberdeen, Surgery<br>Briganti , A; Vita-Salute University San Raffaele; IRCCS Ospedale San Raffaele, Unit of Urology/Division of Oncology, URI<br>Van Hemelrijck, Mieke; King's College London, Translational Oncology and Urology Research (TOUR)
**Primary Subject Heading**:	Urology
Secondary Subject Heading:	Epidemiology, Oncology, Urology
Keywords:	Prostate disease < UROLOGY, ONCOLOGY, Urological tumours < ONCOLOGY

I, the Submitting Author has the right to grant and does grant on behalf of all authors of the Work (as defined in the below author licence), an exclusive licence and/or a non-exclusive licence for contributions from authors who are: i) UK Crown employees; ii) where BMJ has agreed a CC-BY licence shall apply, and/or iii) in accordance with the terms applicable for US Federal Government officers or employees acting as part of their official duties; on a worldwide, perpetual, irrevocable, royalty-free basis to BMJ Publishing Group Ltd ("BMJ") its licensees and where the relevant Journal is co-owned by BMJ to the co-owners of the Journal, to publish the Work in this journal and any other BMJ products and to exploit all rights, as set out in our licence.

The Submitting Author accepts and understands that any supply made under these terms is made by BMJ to the Submitting Author unless you are acting as an employee on behalf of your employer or a postgraduate student of an affiliated institution which is paying any applicable article publishing charge ("APC") for Open Access articles. Where the Submitting Author wishes to make the Work available on an Open Access basis (and intends to pay the relevant APC), the terms of reuse of such Open Access shall be governed by a Creative Commons licence – details of these licences and which Creative Commons licence will apply to this Work are set out in our licence referred to above.

Other than as permitted in any relevant BMJ Author's Self Archiving Policies, I confirm this Work has not been accepted for publication elsewhere, is not being considered for publication elsewhere and does not duplicate material already published. I confirm all authors consent to publication of this Work and authorise the granting of this licence.

For peer review only

A systematic review protocol of diagnostic and prognostic factors in patients with prostate cancer

Author list: Katharina Beyer[a]; Lisa Moris[b]; Michael Lardas[c]; Anna Haire[a]; Francesco Barletta[d] ; Simone Scuderi[d]; Eleni Vradi[e]; Giorgio Gandaglia[d]; Muhammad Imran Omar[f]; Steven Maclennan[f]; Jihong Zong[g]; Bahman Farahmand[h]; Sara J Maclennan[f]; Zsuzsanna Devecseri[i], Alex Asiimwe[e]; Laurence Collette[j]; Anders Bjartell[k], James N'Dow[f]; Alberto Briganti[d,l]; Mieke Van Hemelrijck[a] and PIONEER Consortium*

Corresponding author:

Katharina Beyer,
Translational Oncology and Urology Research (TOUR),
School of Cancer and Pharmaceutical Studies,
3rd Floor, Bermondsey Wing, Guy's Hospital, Great Maze Pond,
London SE1 9RT,
02071885594
katharina.beyer@kcl.ac.uk

Affiliations:

[a] Translational and Oncology Research (TOUR), Faculty of Life Sciences and Medicine, King's College London, London, UK
[b] Department of Urology, University Hospitals Leuven, Leuven, Belgium
[c] Department of Urology, Metropolitan general, Athens, Greece
[d] Unit of Urology/Division of Oncology, URI, IRCCS Ospedale San Raffaele, Milan, Italy
[e] Department of Epidemiology, Bayer AG, Berlin, Germany
[f] Academic Urology Unit, Health Services Research Unit, University of Aberdeen, Aberdeen, UK
[g] Global Epidemiology, Bayer HealthCare Pharmaceuticals Inc., Whippany, USA
[h] Global Epidemiology, Bayer AB, Stockholm, Sweden
[i] Sanofi, Paris, France
[j] EORTC Headquarters, Brussels, Belgium
[k] Department of Translational Medicine, Medical Faculty, Lund University, Lund University, Malmo, Sweden
[l] Department of Urology, University Vita e Salute-San Raffaele, Milan, Italy

Key words: Prostate cancer, diagnostic factor, prognostic factors
Word count of text: 1612

For peer review only

Introduction: As part of the PIONEER Consortium, we will explore which diagnostic and prognostic factors (DPFs) are currently being researched to previously defined clinical and patient-reported outcomes for prostate cancer (PCa).

Methods and analysis: This research project will follow the following four steps: (1) a broad systematic literature review of DPFs for all stages of PCa, covering evidence from 2014 onwards; (2) discussion of systematic review findings by a multidisciplinary expert panel; (3) Risk of bias assessment and applicability with PROBAST (Prediction model Risk Of Bias Assessment Tool) criteria, QUADAS-2 and QUIPS; (4) additional quantitative assessments if required.

Ethics and Dissemination: We aim to develop an online tool to present the DPFs identified in this research and make them available across all stakeholders. There are no ethical implications.

For peer review only

For peer review only - http://bmjopen.bmj.com/site/about/guidelines.xhtml

Strengths and limitations of this study

- This is a novel systematic review to explore relevant diagnostic and prognostic factors for previously defined clinical and patient reported PCa outcomes. It aims to increase the knowledge in this field by focusing on all available quantitative evidence for all stages of PCa.
- A multidisciplinary team including patients, urologists, oncologists, radiation oncologists, methodological experts and pathologists will be involved throughout the study.
- The search was restricted from 2014 onwards, to maintain a pragmatic approach.
- It is possible that our review will not include all articles which have been published in every journal as some may not be accessible.
- We are aware of the limitation of pooling evidence from non-randomised studies, as there could be specific bias inherent within the design. However, detailed assessments of these biases will be conducted.

For peer review only

1. Introduction

Prostate cancer (PCa) is the second most common cancer in men worldwide [1] and accounts for 15% of all cancers diagnosed [2]. Clinically localised PCa is typically characterised by a favourable long-term natural history, where several therapeutic options are available. Moreover, the treatment landscape of advanced and metastatic PCa has changed dramatically in the past decade with the approval of multiple systemic agents, improving patients' survival. PCa is usually suspected based upon the clinical findings of digital rectal examination (DRE) and/or Prostate Specific Antigen (PSA) level [2]. However, which treatment strategy is best or which biomarkers can be used to select patients for specific therapeutic options remains largely uncertain [3]. Multiple diagnostic and prognostic factors (DPFs) are available on top of traditional PSA testing to improve the diagnosis of PCa, such as PCA3, TMPRSS2-ERG fusion, or kallikreins as incorporated in the Phi or 4Kscore test [4-7]. However, the European Association of Urology (EAU) guidelines (2019) currently do not provide recommendations to implement these factors or biomarkers into routine screening programmes due to limited data [2].

The PIONEER Consortium is an international collaboration coordinated by the EAU which aims to establish the best evidence-based management and clinical practice of PCa across all disease stages using the power of big data analytics towards a more outcome-driven, value-based, and patient-centric healthcare system. A key objective is to address one of the major challenges within the context of diagnostic or prognostic biomarkers/factors: the inability to incorporate real-world clinical outcome data into the management of PCa in terms of screening, diagnosis and treatment. Biomarkers can be classified into different types: diagnostic, prognostic, predictive, and therapeutic. A diagnostic biomarker or factor allows

For peer review only - http://bmjopen.bmj.com/site/about/guidelines.xhtml

the early detection of cancer in a non-invasive way and thus the secondary prevention of cancer [8]. A prognostic biomarker or factor is a clinical or biological characteristic that provides information on the likely course of the disease [8]. In the sections below we have used the terms biomarkers and factors interchangeably.

This project investigates which DPFs are available in relation to PIONEER's previously defined core outcomes for PCa [9-13] by evaluating at the evidence published from 2014 onwards as to reflect current medical practice and the '2014 International Society of Urological Pathology (ISUP) Consensus Conference on Grading Patterns and Proposal for a New Grading system'[14]. We specifically aim to assess the strength of the evidence for each DPF and use this information to develop an online search tool on the PIONEER website to be used by all stakeholders

2. Methods and analysis

This research project will follow the following four steps (Figure 1):

(1) We will start with a broad systematic literature-based review of DPFs for all stages of PCa, covering English language publications of human studies from 2014 onwards.

(2) The final list of all available DPFs for which a systematic review is required will then be discussed by a multidisciplinary expert panel.

(3) Each study and systematic review identified through the literature search will be assessed using a risk of bias tool and if applicable the PROBAST (Prediction model Risk Of Bias ASsessment Tool) criteria.

For peer review only - http://bmjopen.bmj.com/site/about/guidelines.xhtml

(4) For those studies with an overall low score on risk of bias and low concerns for the applicability of their results, we will use the Classification from the Oxford Centre for Evidence Based Medicine to define whether an additional quantitative assessment is required.

2.1. Stage 1: Systematic review

The systematic review will be conducted in accordance with the Preferred Reporting Items for Systematic Reviews and MetaAnalyses (PRISMA) guidelines [15 16].

2.1.1 Search methods and identification of studies:

The literature search has been developed by an experienced Information Scientist. We will search for quantitative observational studies which assess either diagnostic or prognostic factors. The search strategy is shown in Table 1.

The eligibility will be independently checked by at least two researchers. Conflicts will be solved by discussion or consulting an additional author. The abstract and full-text screening will be conducted in duplicate, and results will be compared and shared with the core group involved in the research project. The EAU guidelines will also be hand-searched to identify DPFs [2].

2.1.2. Eligibility criteria

Types of studies: We will include quantitative studies only. Studies included in this systematic review will be limited to those which are published between January 2014 and January 2020, so that the project is pragmatic. Qualitative studies, narrative literature

For peer review only - http://bmjopen.bmj.com/site/about/guidelines.xhtml

reviews and commentary pieces will be excluded. Single studies with fewer than 50 participants will be excluded since a small patient number is unlikely to influence practice due to lack of power and risk of selection bias in the study. In addition, the test is unlikely to be ready for clinical usage. Studies which are not published in English language or are out of our defined timeframe will also be excluded. We believe that DPFs which are relevant to clinical practice and are developed outside of the proposed timeframe will either be captured in the EAU guidelines [2] or are already included in the evidence reviewed due to high relevance.

Types of participants: The participants are adult men (≥18 years of age) diagnosed with:

a) Localised PCa: ≤T2cN0M0 with no treatment prior to their primary treatment for PCa (except for neoadjuvant androgen deprivation therapy [ADT] preceding radiotherapy). Studies in which locally advanced or metastatic disease (T3-T4 N+ or M+) accounted for >10% of their participants will be excluded unless the localised participants are reported separately.
b) Locally advanced PCa: T3 or T4 and/or N1.
c) Metastatic PCa: any T, any N, M1 a-c. Studies in which local disease (≤ any T, any N, M0) accounted for >10% of their participants will be excluded, unless the metastatic participants are reported separately.
d) nmCRPC: any T, N, M0 and castration-resistance defined as a castrate serum testosterone < 50 ng/dL or 1.7 nmol/L plus either biochemical or radiological progression.

For peer review only - http://bmjopen.bmj.com/site/about/guidelines.xhtml

Types of interventions: Interventions considered for this systematic review will be all treatments supported by the 2019 EAU guidelines [2] for localised, locally advanced, metastatic and non-metastatic castration resistant PCa.

Data extraction: Data will be extracted by at least two reviewers and checked by a third reviewer following the Critical Appraisal and Data Extraction for systematic reviews of prediction modelling studies (CHARMS PF Checklist)[17 18].

2.2. Stage 2: Assessment of Stage 1 output with expert panel to identify the individual topics for systematic reviews.

An expert panel of urologists, radiologists, radiation oncologist, oncologist, methodologist and pathologists will review the extracted factors to discuss if any DPFs are missing. If no DPFs are missing, the review team will assess the quality of the identified studies for each DPF systematic review using the PROBAST (Prediction model Risk Of Bias ASsessment Tool) criteria [19 20].

2.3. Stage 3: Risk of Bias assessment of individual articles using PROBAST

Each study and systematic review identified through the literature search will be assessed using PROBAST (Prediction model Risk Of Bias ASsessment Tool) criteria [19]. PROBAST is a tool to assess the risk of bias as well as the applicability of diagnostic and prognostic prediction models. For studies that will not meet the PROBAST criteria, we will use QUADAS-2 [21] for diagnostic factors accuracy studies and QUIPS [22] for prognostic factors studies.

For peer review only

2.4. Stage 4: Quantitative assessment of individual articles using meta-analytic techniques

We are well aware of the limitation of pooling evidence from non-randomised studies, as there could be specific bias inherent within the design. We will be very cautious while pooling evidence from non-randomised studies. For those studies with an overall low score on risk of bias and low on concerns for applicability, we will use the Classification from the Oxford Centre for Evidence Based Medicine to define whether an additional quantitative assessment is required.

1. If there is Level 1a (SR of inception cohort studies validated in different populations or Level 1 diagnostic studies from different clinical centres), we do not do a meta-analysis.
2. No Level 1a but >2 studies which apply to 1b (Prognostic: individual inception cohort study with > 80% follow-up; Clinical Decision Rule (CDR) validated in a single population; Diagnostic: Validating cohort study with good reference standards; or CDR tested within one clinical centre) or 1c (Prognostic: All or none case-series; Diagnostic: Absolute specificity is so high that a positive result rules-in the diagnosis (SpPins) and diagnostic finding whose Sensitivity is so high that a Negative result rules-out the diagnosis (SnNouts)), we will perform a meta-analysis.

In the situation where we are not able to perform additional meta-analysis due to low quality of the assessed papers, we will add an additional stage to the review and discuss the data with the DPF PIONEER expert panel. We will aim to provide recommendations for researchers to improve the quality of future studies and enable the conduct of meta-

For peer review only - http://bmjopen.bmj.com/site/about/guidelines.xhtml

analyses. We will follow the methodology developed by the Joanna Briggs Institute guidelines [23] and the framework by Arksey and O'Malley [24].

2.5. Patient and Public Involvement

PIONEER brings together 32 key stakeholders in prostate cancer research and clinical care from across 9 countries. Consortium members are drawn from academic institutions, European organisations, patient advocacy groups, clinicians, and pharmaceutical companies, as well as regulatory agencies, experts in legal data management, economics and ethics, and information and technology specialists. Hence, the patients and their family members are an integral part of all research conducted by the PIONEER Consortium.

3. Ethics and Dissemination

No additional ethical approval is required as the work will rely on published publicly available study data.

The findings of these systematic reviews will be exported into an online search tool to ensure wide applicability of the study findings. More specifically, this online search tool will produce evidence-based recommendations so that these could be used by researchers, clinicians and experts in the field. The tool will be designed so that stakeholders can access up to date available evidence (and view the quality of the studies published) when developing new DPFs or setting up clinical trials. To ensure sustainability of this tool, PIONEER and the EAU aim to update the systematic reviews described above on a regular basis to reflect the latest available research on DPFs for PCa.

For peer review only

a. Contributorship statement: Design of protocol: LM, ML, AH, FB, SS, EV, GG, MIO, SM, JZ, BF, SJM, ZD, AA, LC, AB, JND, AB, MvH. Draft of manuscript: KB, MIO, JND, MVH. Final approval of manuscript: LM, ML, AH, FB, SS, EV, GG, MIO, SM, JZ, BF, SJM, ZD, AA, LC, AB, JND, AB, MvH

b. Competing interests: None

c. Funding: The IMI2 Joint Undertaking under grant agreement No. 777492

d. Data sharing statement: Data are available in a public, open access repository

For peer review only

For peer review only - http://bmjopen.bmj.com/site/about/guidelines.xhtml

References

1. Bray F, Ferlay J, Soerjomataram I, et al. Global cancer statistics 2018: GLOBOCAN estimates of incidence and mortality worldwide for 36 cancers in 185 countries. *CA: a cancer journal for clinicians* 2018;68(6):394-424. doi: 10.3322/caac.21492 [published Online First: 2018/09/13]
2. Mottet N, Bellmunt J, Bolla M, et al. EAU-ESTRO-SIOG Guidelines on Prostate Cancer. Part 1: Screening, Diagnosis, and Local Treatment with Curative Intent. *European urology* 2017;71(4):618-29. doi: 10.1016/j.eururo.2016.08.003 [published Online First: 2016/08/30]
3. Filella X, Fernandez-Galan E, Fernandez Bonifacio R, et al. Emerging biomarkers in the diagnosis of prostate cancer. *Pharmacogenomics and personalized medicine* 2018;11:83-94. doi: 10.2147/pgpm.S136026 [published Online First: 2018/05/31]
4. Vedder MM, de Bekker-Grob EW, Lilja HG, et al. The added value of percentage of free to total prostate-specific antigen, PCA3, and a kallikrein panel to the ERSPC risk calculator for prostate cancer in prescreened men. *European urology* 2014;66(6):1109-15. doi: 10.1016/j.eururo.2014.08.011 [published Online First: 2014/08/30]
5. Leyten GH, Hessels D, Jannink SA, et al. Prospective multicentre evaluation of PCA3 and TMPRSS2-ERG gene fusions as diagnostic and prognostic urinary biomarkers for prostate cancer. *European urology* 2014;65(3):534-42. doi: 10.1016/j.eururo.2012.11.014 [published Online First: 2012/12/04]
6. Boegemann M, Stephan C, Cammann H, et al. The percentage of prostate-specific antigen (PSA) isoform [-2]proPSA and the Prostate Health Index improve the diagnostic accuracy for clinically relevant prostate cancer at initial and repeat biopsy compared with total PSA and percentage free PSA in men aged </=65 years. *BJU international* 2016;117(1):72-9. doi: 10.1111/bju.13139 [published Online First: 2015/03/31]
7. Bryant RJ, Sjoberg DD, Vickers AJ, et al. Predicting high-grade cancer at ten-core prostate biopsy using four kallikrein markers measured in blood in the ProtecT study. *Journal of the National Cancer Institute* 2015;107(7) doi: 10.1093/jnci/djv095 [published Online First: 2015/04/12]
8. Carlomagno N, Incollingo P, Tammaro V, et al. Diagnostic, Predictive, Prognostic, and Therapeutic Molecular Biomarkers in Third Millennium: A Breakthrough in Gastric Cancer. *BioMed research international* 2017;2017:7869802. doi: 10.1155/2017/7869802 [published Online First: 2017/11/03]
9. Beyer K ML, Lardas M, Maclennan S, Monagas S, Antunes-Lopes T, et al. PIONEER's systematic review of outcomes reported in effectiveness trials for interventions in locally advanced prostate cancer. 11th European Multidisciplinary Congress on Urological Cancers. 11th European Multidisciplinary Congress on Urological Cancers. Vienna, 2019.
10. Beyer K MS, Lardas M, Moris L, Omar M, Flaherty S. PIONEER's update and integration of a localised prostate cancer core outcome set for effectiveness trials and a standard set for clinical practice. Annual Meeting of the European Association of Urology. Annual Meeting of the European Association of Urology. Barcelona2019, 2019.
11. Beyer K MS, Lardas M, Moris L, Vradi E, Picozzi M, et al. . PIONEER's update and integration of a metastatic Prostate Cancer Core Outcome Set. ICHOM 2019. Rotterdam, 2019.
12. Beyer K MS, Lardas M, Moris L, Omar M, MacLennan S, et al. PIONEER's update and integration of a localised prostate cancer core outcome set for effectiveness trials and a standard set for clinical practice. 11th European Multidisciplinary Congress on Urological Cancers. 11th European Multidisciplinary Congress on Urological Cancers. Vienna, 2019.
13. Beyer K MS, Lardas M, Moris L, Vradi E, Omar, I, et al PIONEER's systematic review of outcomes in RCTs of men with non-metastatic castration resistant prostate cancer. Is there a need for a core outcome set? Annual Meeting of the European Association of Urology. Amsterdam, 2020.
14. Epstein JI, Egevad L, Amin MB, et al. The 2014 International Society of Urological Pathology (ISUP) Consensus Conference on Gleason Grading of Prostatic Carcinoma: Definition of

For peer review only - http://bmjopen.bmj.com/site/about/guidelines.xhtml

Grading Patterns and Proposal for a New Grading System. *The American journal of surgical pathology* 2016;40(2):244-52. doi: 10.1097/pas.0000000000000530 [published Online First: 2015/10/23]
15. Moher D, Liberati A, Tetzlaff J, et al. Preferred reporting items for systematic reviews and meta-analyses: the PRISMA statement. *PLoS medicine* 2009;6(7):e1000097-e97. doi: 10.1371/journal.pmed.1000097
16. Higgins JPT TJ, Chandler J, Cumpston M, Li T, Page MJ, Welch VA Cochrane Handbook for Systematic Reviews of Interventions. Chichester (UK): Cochrane, 2019.
17. Moons KGM, de Groot JAH, Bouwmeester W, et al. Critical Appraisal and Data Extraction for Systematic Reviews of Prediction Modelling Studies: The CHARMS Checklist. *PLOS Medicine* 2014;11(10):e1001744. doi: 10.1371/journal.pmed.1001744
18. Riley RD, Moons KGM, Snell KIE, et al. A guide to systematic review and meta-analysis of prognostic factor studies. *BMJ (Clinical research ed)* 2019;364:k4597. doi: 10.1136/bmj.k4597 [published Online First: 2019/02/01]
19. Wolff RF, Moons KGM, Riley RD, et al. PROBAST: A Tool to Assess the Risk of Bias and Applicability of Prediction Model Studies. *Annals of Internal Medicine* 2019;170(1):51-58. doi: 10.7326/M18-1376
20. Moons KGM, Wolff RF, Riley RD, et al. PROBAST: A Tool to Assess Risk of Bias and Applicability of Prediction Model Studies: Explanation and Elaboration. *Ann Intern Med* 2019;170(1):W1-w33. doi: 10.7326/m18-1377 [published Online First: 2019/01/01]
21. Whiting PF, Rutjes AW, Westwood ME, et al. QUADAS-2: a revised tool for the quality assessment of diagnostic accuracy studies. *Ann Intern Med* 2011;155(8):529-36. doi: 10.7326/0003-4819-155-8-201110180-00009 [published Online First: 2011/10/19]
22. Hayden JA, van der Windt DA, Cartwright JL, et al. Assessing bias in studies of prognostic factors. *Ann Intern Med* 2013;158(4):280-6. doi: 10.7326/0003-4819-158-4-201302190-00009 [published Online First: 2013/02/20]
23. Peters MD, Godfrey CM, Khalil H, et al. Guidance for conducting systematic scoping reviews. *Int J Evid Based Healthc* 2015;13(3):141-6. doi: 10.1097/xeb.0000000000000050 [published Online First: 2015/07/03]
24. Arksey H, O'Malley L. Scoping studies: towards a methodological framework. *International Journal of Social Research Methodology* 2005;8(1):19-32. doi: 10.1080/1364557032000119616

For peer review only

For peer review only - http://bmjopen.bmj.com/site/about/guidelines.xhtml

Tables and Figures

Table 1: Search strategy

Database: OVID Medline Epub Ahead of Print, In-Process & Other Non-Indexed Citations, Ovid MEDLINE(R) Daily and Ovid MEDLINE(R) 1946 to Present, Embase <1974 to 2020 January 28>, EBM Reviews - Cochrane Database of Systematic Reviews <2005 to January 21, 2020>

Search Strategy:

----

1 exp *Prostatic Neoplasms/ (262435)

2 exp *prostate cancer/ (245472)

3 (prostat* adj2 (cancer* or carcinoma* or malignan* or tumor* or tumour* or neoplas* or adenocarcinoma* or adenoma*)).tw. (332251)

4 or/1-3 (366427)

5 ((diagnostic or prognos* or predict*) adj10 (biomarker or biomarkers or factor or factors)).tw,kw. (717487)

6 ((diagnostic or prognos* or predict*) adj10 (Oncotype Dx Prostate or Prolaris or Decipher or Decipher PORTOS or ProMark)).tw,kw. (458)

7 5 or 6 (717869)

8 4 and 7 (17456)

9 limit 8 to english language [Limit not valid in CDSR; records were retained] (16484)

10 limit 9 to yr="2014 -Current" (8417)

11 conference abstract.pt. or Congresses as Topic/ or Conference Review.pt. or "Journal: Conference Abstract".pt. (3815712)

12 10 not 11 (5902)

13 (exp animals/ or exp animal/ or exp nonhuman/ or exp animal experiment/ or animal model/ or animal tissue/ or non human/ or (rat or rats or mice or mouse or swine or porcine or murine or sheep or lambs or pigs or piglets or rabbit or rabbits or cat or cats or dog or dogs or cattle or bovine or monkey or monkeys or trout or marmoset$1).ti.) not (humans/ or human/ or human experiment/ or (human* or men or women or patients or subjects).tw.) (10251935)

14 12 not 13 (5882)

15 note/ or editorial/ or letter/ or Comment/ or news/ or (note or editorial or letter or Comment or news).pt. (4565255)

16 14 not 15 (5811)

17 (child/ or Pediatrics/ or Adolescent/ or Infant/ or adolescence/ or newborn/ or (baby or babies or child or children or pediatric* or paediatric* or peadiatric* or infant* or infancy or neonat* or

For peer review only

For peer review only - http://bmjopen.bmj.com/site/about/guidelines.xhtml

newborn* or new born* or adolescen* or preschool or pre-school or toddler*).tw.) not (adult/ or aged/ or (aged or adult* or elder* or senior* or men or women).tw.) (4146377)

18 16 not 17 (5794)

19 18 use ppez,oemezd (5788)

20 10 use coch (6)

21 19 or 20 (5794)

22 remove duplicates from 21 (3140)

Figure 1: Overview of four stage process

For peer review only

Figure 1: Overview of four stage process

Workflow	Task
Stage 1.	Broad literature-based systematic review of diagnostic and prognostic factors (DPFs) for all stages of prostate cancer from 2014 onwards (English only; humans).<br>• Extract data from the included studies following the CHARMS-PF guideline.
Stage 2.	Discussion of systematic review findings by a multidisciplinary expert panel<br>• Review the list of included studies
Stage 3.	Risk of Bias Assessment and applicability of individual studies using PROBAST, QUIPS and QUADAS-2
Stage 4.	Quantitative assessment of individual articles using meta-analytic techniques:<br>• If PROBAST indicates low risk of bias and low concerns for applicability:<br>Oxford Classification Centre for Evidence Based Medicine:<br>1. If there is Level 1a (SR of inception cohort studies validated in different populations or Level 1 diagnostic studies from different clinical centres), we do not do a meta-analysis<br>2. No Level 1a but >2 studies which apply to 1b (Prognostic: individual inception cohort study with > 80% follow-up; CDR" validated in a single population; Diagnostic: Validating cohort study with good reference standards; or CDR" tested within one clinical centre) or 1c (Prognostic: All or none case-series; diagnostic: Absolute SpPins and SnNouts), we do a meta-analysis
Final aim:	Develop online PIONEER Online Search Tool for DPFs

For peer review only

BMJ Open

A systematic review protocol of diagnostic and prognostic factors in patients with prostate cancer

Journal:	*BMJ Open*
Manuscript ID	bmjopen-2020-040531.R2
Article Type:	Protocol
Date Submitted by the Author:	22-Dec-2020
Complete List of Authors:	Beyer, Katharina; King's College London, Translational Oncology and Urology Research (TOUR)<br>Moris, Lisa; KU Leuven University Hospitals Leuven, Department of Urology<br>Lardas, Michael; Metropolitan Hospital Athens, Department of Urology<br>Haire, Anna ; King's College London, Translational Oncology and Urology Research (TOUR)<br>Barletta, Francesco; IRCCS Ospedale San Raffaele, Unit of Urology/Division of Oncology, URI<br>Scuderi, Simone; IRCCS Ospedale San Raffaele, Unit of Urology/Division of Oncology, URI<br>Vradi, Eleni; Bayer Pharma AG, Medical Affairs and PV<br>Gandaglia, Giorgio; IRCCS Ospedale San Raffaele, Unit of Urology/Division of Oncology, URI<br>Omar, Muhammad; University of Aberdeen, Academic Urology Unit, Health Services Research Unit<br>MacLennan, Steven; University of Aberdeen, Academic Urology Unit, Health Services Research Unit<br>Zong, Jihong; Bayer U.S. , Epidemiology<br>Farahmand, Bahman; Bayer AG<br>Maclennan, Sara; University of Aberdeen, Academic Urology Unit, Health Services Research Unit<br>Devecseri, Zsuzsanna; Sanofi SA<br>Asiimwe, Alex; Bayer Pharma AG, Medical Affairs and PV<br>Collette, Laurence ; EORTC<br>Bjartell, Anders; Skåne University Hospital,<br>NDow, James; University of Aberdeen, Surgery<br>Briganti , A; Vita-Salute University San Raffaele; IRCCS Ospedale San Raffaele, Unit of Urology/Division of Oncology, URI<br>Van Hemelrijck, Mieke; King's College London, Translational Oncology and Urology Research (TOUR)
**Primary Subject Heading**:	Urology
Secondary Subject Heading:	Epidemiology, Oncology, Urology
Keywords:	Prostate disease < UROLOGY, ONCOLOGY, Urological tumours < ONCOLOGY

I, the Submitting Author has the right to grant and does grant on behalf of all authors of the Work (as defined in the below author licence), an exclusive licence and/or a non-exclusive licence for contributions from authors who are: i) UK Crown employees; ii) where BMJ has agreed a CC-BY licence shall apply, and/or iii) in accordance with the terms applicable for US Federal Government officers or employees acting as part of their official duties; on a worldwide, perpetual, irrevocable, royalty-free basis to BMJ Publishing Group Ltd ("BMJ") its licensees and where the relevant Journal is co-owned by BMJ to the co-owners of the Journal, to publish the Work in this journal and any other BMJ products and to exploit all rights, as set out in our licence.

The Submitting Author accepts and understands that any supply made under these terms is made by BMJ to the Submitting Author unless you are acting as an employee on behalf of your employer or a postgraduate student of an affiliated institution which is paying any applicable article publishing charge ("APC") for Open Access articles. Where the Submitting Author wishes to make the Work available on an Open Access basis (and intends to pay the relevant APC), the terms of reuse of such Open Access shall be governed by a Creative Commons licence – details of these licences and which Creative Commons licence will apply to this Work are set out in our licence referred to above.

Other than as permitted in any relevant BMJ Author's Self Archiving Policies, I confirm this Work has not been accepted for publication elsewhere, is not being considered for publication elsewhere and does not duplicate material already published. I confirm all authors consent to publication of this Work and authorise the granting of this licence.

For peer review only

A systematic review protocol of diagnostic and prognostic factors in patients with prostate cancer

*Author list: Katharina Beyer[a]; Lisa Moris[b]; Michael Lardas[c]; Anna Haire[a]; Francesco Barletta[d] ; Simone Scuderi[d]; Eleni Vradi[e]; Giorgio Gandaglia[d]; Muhammad Imran Omar[f]; Steven Maclennan[f]; Jihong Zong[g]; Bahman Farahmand[h]; Sara J Maclennan[f]; Zsuzsanna Devecseri[i], Alex Asiimwe[e]; Laurence Collette[j]; Anders Bjartell[k], James N'Dow[f]; Alberto Briganti[d,l]; Mieke Van Hemelrijck[a]*and PIONEER Consortium*

Corresponding author:

Katharina Beyer,
Translational Oncology and Urology Research (TOUR),
School of Cancer and Pharmaceutical Studies,
3rd Floor, Bermondsey Wing, Guy's Hospital, Great Maze Pond,
London SE1 9RT,
02071885594
katharina.beyer@kcl.ac.uk

Affiliations:

[a] Translational and Oncology Research (TOUR), Faculty of Life Sciences and Medicine, King's College London, London, UK
[b] Department of Urology, University Hospitals Leuven, Leuven, Belgium
[c] Department of Urology, Metropolitan general, Athens, Greece
[d] Unit of Urology/Division of Oncology, URI, IRCCS Ospedale San Raffaele, Milan, Italy
[e] Department of Epidemiology, Bayer AG, Berlin, Germany
[f] Academic Urology Unit, Health Services Research Unit, University of Aberdeen, Aberdeen, UK
[g] Global Epidemiology, Bayer HealthCare Pharmaceuticals Inc., Whippany, USA
[h] Global Epidemiology, Bayer AB, Stockholm, Sweden
[i] Sanofi, Paris, France
[j] EORTC Headquarters, Brussels, Belgium
[k] Department of Translational Medicine, Medical Faculty, Lund University, Lund University, Malmo, Sweden
[l] Department of Urology, University Vita e Salute-San Raffaele, Milan, Italy

Key words: Prostate cancer, diagnostic factor, prognostic factors
Word count of text: 1612

For peer review only

Introduction: As part of the PIONEER Consortium, we will explore which diagnostic and prognostic factors (DPFs) are currently being researched to previously defined clinical and patient-reported outcomes for prostate cancer (PCa).

Methods and analysis: This research project will follow the following four steps: (1) a broad systematic literature review of DPFs for all stages of PCa, covering evidence from 2014 onwards; (2) discussion of systematic review findings by a multidisciplinary expert panel; (3) Risk of bias assessment and applicability with PROBAST (Prediction model Risk Of Bias Assessment Tool) criteria, QUADAS-2 and QUIPS; (4) additional quantitative assessments if required.

Ethics and Dissemination: We aim to develop an online tool to present the DPFs identified in this research and make them available across all stakeholders. There are no ethical implications.

For peer review only

For peer review only - http://bmjopen.bmj.com/site/about/guidelines.xhtml

Strengths and limitations of this study

- This is a novel systematic review to explore relevant diagnostic and prognostic factors for previously defined clinical and patient reported PCa outcomes. It aims to increase the knowledge in this field by focusing on all available quantitative evidence for all stages of PCa.
- A multidisciplinary team including patients, urologists, oncologists, radiation oncologists, methodological experts and pathologists will be involved throughout the study.
- The search was restricted from 2014 onwards, to maintain a pragmatic approach.
- It is possible that our review will not include all articles which have been published in every journal as some may not be accessible.
- We are aware of the limitation of pooling evidence from non-randomised studies, as there could be specific bias inherent within the design. However, detailed assessments of these biases will be conducted.

For peer review only

For peer review only - http://bmjopen.bmj.com/site/about/guidelines.xhtml

1. Introduction

Prostate cancer (PCa) is the second most common cancer in men worldwide [1] and accounts for 15% of all cancers diagnosed [2]. Clinically localised PCa is typically characterised by a favourable long-term natural history, where several therapeutic options are available. Moreover, the treatment landscape of advanced and metastatic PCa has changed dramatically in the past decade with the approval of multiple systemic agents, improving patients' survival. PCa is usually suspected based upon the clinical findings of digital rectal examination (DRE) and/or Prostate Specific Antigen (PSA) level [2]. However, which treatment strategy is best or which biomarkers can be used to select patients for specific therapeutic options remains largely uncertain [3]. Multiple diagnostic and prognostic factors (DPFs) are available on top of traditional PSA testing to improve the diagnosis of PCa, such as PCA3, TMPRSS2-ERG fusion, or kallikreins as incorporated in the Phi or 4Kscore test [4-7]. However, the European Association of Urology (EAU) guidelines (2019) currently do not provide recommendations to implement these factors or biomarkers into routine screening programmes due to limited data [2].

The PIONEER Consortium is an international collaboration coordinated by the EAU which aims to establish the best evidence-based management and clinical practice of PCa across all disease stages using the power of big data analytics towards a more outcome-driven, value-based, and patient-centric healthcare system. A key objective is to address one of the major challenges within the context of diagnostic or prognostic biomarkers/factors: the inability to incorporate real-world clinical outcome data into the management of PCa in terms of screening, diagnosis and treatment. Biomarkers can be classified into different types: diagnostic, prognostic, predictive, and therapeutic. A diagnostic biomarker or factor allows

For peer review only - http://bmjopen.bmj.com/site/about/guidelines.xhtml

the early detection of cancer in a non-invasive way and thus the secondary prevention of cancer [8]. A prognostic biomarker or factor is a clinical or biological characteristic that provides information on the likely course of the disease [8]. In the sections below we have used the terms biomarkers and factors interchangeably.

This project investigates which DPFs are available in relation to PIONEER's previously defined core outcomes for PCa [9-13] by evaluating at the evidence published from 2014 onwards as to reflect current medical practice and the '2014 International Society of Urological Pathology (ISUP) Consensus Conference on Grading Patterns and Proposal for a New Grading system'[14]. We specifically aim to assess the strength of the evidence for each DPF and use this information to develop an online search tool on the PIONEER website to be used by all stakeholders

2. Methods and analysis

This research project will follow the following four steps (Figure 1):

(1) We will start with a broad systematic literature-based review of DPFs for all stages of PCa, covering English language publications of human studies from 2014 onwards.

(2) The final list of all available DPFs for which a systematic review is required will then be discussed by a multidisciplinary expert panel.

(3) Each study and systematic review identified through the literature search will be assessed using a risk of bias tool and if applicable the PROBAST (Prediction model Risk Of Bias ASsessment Tool) criteria.

For peer review only - http://bmjopen.bmj.com/site/about/guidelines.xhtml

(4) For those studies with an overall low score on risk of bias and low concerns for the applicability of their results, we will use the Classification from the Oxford Centre for Evidence Based Medicine to define whether an additional quantitative assessment is required.

2.1. Stage 1: Systematic review

The systematic review will be conducted in accordance with the Preferred Reporting Items for Systematic Reviews and MetaAnalyses (PRISMA) guidelines [15 16].

2.1.1 Search methods and identification of studies:

The literature search has been developed by an experienced Information Scientist. We will search for quantitative observational studies which assess either diagnostic or prognostic factors. The search strategy is shown in Table 1.

The eligibility will be independently checked by at least two researchers. Conflicts will be solved by discussion or consulting an additional author. The abstract and full-text screening will be conducted in duplicate, and results will be compared and shared with the core group involved in the research project. The EAU guidelines will also be hand-searched to identify DPFs [2].

2.1.2. Eligibility criteria

Types of studies: We will include quantitative studies only. Studies included in this systematic review will be limited to those which are published between January 2014 and January 2020, so that the project is pragmatic. Qualitative studies, narrative literature

For peer review only - http://bmjopen.bmj.com/site/about/guidelines.xhtml

reviews and commentary pieces will be excluded. Single studies with fewer than 50 participants will be excluded since a small patient number is unlikely to influence practice due to lack of power and risk of selection bias in the study. In addition, the test is unlikely to be ready for clinical usage. Studies which are not published in English language or are out of our defined timeframe will also be excluded. We believe that DPFs which are relevant to clinical practice and are developed outside of the proposed timeframe will either be captured in the EAU guidelines [2] or are already included in the evidence reviewed due to high relevance.

Types of participants: The participants are adult men (≥18 years of age) diagnosed with:

a) Localised PCa: ≤T2cN0M0 with no treatment prior to their primary treatment for PCa (except for neoadjuvant androgen deprivation therapy [ADT] preceding radiotherapy). Studies in which locally advanced or metastatic disease (T3-T4 N+ or M+) accounted for >10% of their participants will be excluded unless the localised participants are reported separately.
b) Locally advanced PCa: T3 or T4 and/or N1.
c) Metastatic PCa: any T, any N, M1 a-c. Studies in which local disease (≤ any T, any N, M0) accounted for >10% of their participants will be excluded, unless the metastatic participants are reported separately.
d) nmCRPC: any T, N, M0 and castration-resistance defined as a castrate serum testosterone < 50 ng/dL or 1.7 nmol/L plus either biochemical or radiological progression.

For peer review only - http://bmjopen.bmj.com/site/about/guidelines.xhtml

Types of interventions: Interventions considered for this systematic review will be all treatments supported by the 2019 EAU guidelines [2] for localised, locally advanced, metastatic and non-metastatic castration resistant PCa.

Data extraction: Data will be extracted by at least two reviewers and checked by a third reviewer following the Critical Appraisal and Data Extraction for systematic reviews of prediction modelling studies (CHARMS PF Checklist)[17 18].

2.2. Stage 2: Assessment of Stage 1 output with expert panel to identify the individual topics for systematic reviews.

An expert panel of urologists, radiologists, radiation oncologist, oncologist, methodologist and pathologists will review the extracted factors to discuss if any DPFs are missing. If no DPFs are missing, the review team will assess the quality of the identified studies for each DPF systematic review using the PROBAST (Prediction model Risk Of Bias ASsessment Tool) criteria [19 20].

2.3. Stage 3: Risk of Bias assessment of individual articles using PROBAST

Each study and systematic review identified through the literature search will be assessed using PROBAST (Prediction model Risk Of Bias ASsessment Tool) criteria [19]. PROBAST is a tool to assess the risk of bias as well as the applicability of diagnostic and prognostic prediction models. For studies that will not meet the PROBAST criteria, we will use QUADAS-2 [21] for diagnostic factors accuracy studies and QUIPS [22] for prognostic factors studies.

For peer review only - http://bmjopen.bmj.com/site/about/guidelines.xhtml

2.4. Stage 4: Quantitative assessment of individual articles using meta-analytic techniques

We are well aware of the limitation of pooling evidence from non-randomised studies, as there could be specific bias inherent within the design. We will be very cautious while pooling evidence from non-randomised studies. For those studies with an overall low score on risk of bias and low on concerns for applicability, we will use the Classification from the Oxford Centre for Evidence Based Medicine to define whether an additional quantitative assessment is required.

1. If there is Level 1a (SR of inception cohort studies validated in different populations or Level 1 diagnostic studies from different clinical centres), we do not do a meta-analysis.
2. No Level 1a but >2 studies which apply to 1b (Prognostic: individual inception cohort study with > 80% follow-up; Clinical Decision Rule (CDR) validated in a single population; Diagnostic: Validating cohort study with good reference standards; or CDR tested within one clinical centre) or 1c (Prognostic: All or none case-series; Diagnostic: Absolute specificity is so high that a positive result rules-in the diagnosis (SpPins) and diagnostic finding whose Sensitivity is so high that a Negative result rules-out the diagnosis (SnNouts)), we will perform a meta-analysis.

In the situation where we are not able to perform additional meta-analysis due to low quality of the assessed papers, we will add an additional stage to the review and discuss the data with the DPF PIONEER expert panel. We will aim to provide recommendations for researchers to improve the quality of future studies and enable the conduct of meta-

For peer review only - http://bmjopen.bmj.com/site/about/guidelines.xhtml

analyses. We will follow the methodology developed by the Joanna Briggs Institute guidelines [23] and the framework by Arksey and O'Malley [24].

2.5. Patient and Public Involvement

PIONEER brings together 32 key stakeholders in prostate cancer research and clinical care from across 9 countries. Consortium members are drawn from academic institutions, European organisations, patient advocacy groups, clinicians, and pharmaceutical companies, as well as regulatory agencies, experts in legal data management, economics and ethics, and information and technology specialists. Hence, the patients and their family members are an integral part of all research conducted by the PIONEER Consortium.

3. Ethics and Dissemination

No additional ethical approval is required as the work will rely on published publicly available study data.

The findings of these systematic reviews will be exported into an online search tool to ensure wide applicability of the study findings. More specifically, this online search tool will produce evidence-based recommendations so that these could be used by researchers, clinicians and experts in the field. The tool will be designed so that stakeholders can access up to date available evidence (and view the quality of the studies published) when developing new DPFs or setting up clinical trials. To ensure sustainability of this tool, PIONEER and the EAU aim to update the systematic reviews described above on a regular basis to reflect the latest available research on DPFs for PCa.

For peer review only - http://bmjopen.bmj.com/site/about/guidelines.xhtml

a. Contributorship statement: Design of protocol: LM, ML, AH, FB, SS, EV, GG, MIO, SM, JZ, BF, SJM, ZD, AA, LC, AB, JND, AB, MvH. Draft of manuscript: KB, MIO, JND, MVH. Final approval of manuscript: LM, ML, AH, FB, SS, EV, GG, MIO, SM, JZ, BF, SJM, ZD, AA, LC, AB, JND, AB, MvH

b. Competing interests: None

c. Funding: The IMI2 Joint Undertaking under grant agreement No. 777492

d. Data sharing statement: Data will be available in a public, open access repository

For peer review only - http://bmjopen.bmj.com/site/about/guidelines.xhtml

References

1. Bray F, Ferlay J, Soerjomataram I, et al. Global cancer statistics 2018: GLOBOCAN estimates of incidence and mortality worldwide for 36 cancers in 185 countries. *CA: a cancer journal for clinicians* 2018;68(6):394-424. doi: 10.3322/caac.21492 [published Online First: 2018/09/13]
2. Mottet N, Bellmunt J, Bolla M, et al. EAU-ESTRO-SIOG Guidelines on Prostate Cancer. Part 1: Screening, Diagnosis, and Local Treatment with Curative Intent. *European urology* 2017;71(4):618-29. doi: 10.1016/j.eururo.2016.08.003 [published Online First: 2016/08/30]
3. Filella X, Fernandez-Galan E, Fernandez Bonifacio R, et al. Emerging biomarkers in the diagnosis of prostate cancer. *Pharmacogenomics and personalized medicine* 2018;11:83-94. doi: 10.2147/pgpm.S136026 [published Online First: 2018/05/31]
4. Vedder MM, de Bekker-Grob EW, Lilja HG, et al. The added value of percentage of free to total prostate-specific antigen, PCA3, and a kallikrein panel to the ERSPC risk calculator for prostate cancer in prescreened men. *European urology* 2014;66(6):1109-15. doi: 10.1016/j.eururo.2014.08.011 [published Online First: 2014/08/30]
5. Leyten GH, Hessels D, Jannink SA, et al. Prospective multicentre evaluation of PCA3 and TMPRSS2-ERG gene fusions as diagnostic and prognostic urinary biomarkers for prostate cancer. *European urology* 2014;65(3):534-42. doi: 10.1016/j.eururo.2012.11.014 [published Online First: 2012/12/04]
6. Boegemann M, Stephan C, Cammann H, et al. The percentage of prostate-specific antigen (PSA) isoform [-2]proPSA and the Prostate Health Index improve the diagnostic accuracy for clinically relevant prostate cancer at initial and repeat biopsy compared with total PSA and percentage free PSA in men aged </=65 years. *BJU international* 2016;117(1):72-9. doi: 10.1111/bju.13139 [published Online First: 2015/03/31]
7. Bryant RJ, Sjoberg DD, Vickers AJ, et al. Predicting high-grade cancer at ten-core prostate biopsy using four kallikrein markers measured in blood in the ProtecT study. *Journal of the National Cancer Institute* 2015;107(7) doi: 10.1093/jnci/djv095 [published Online First: 2015/04/12]
8. Carlomagno N, Incollingo P, Tammaro V, et al. Diagnostic, Predictive, Prognostic, and Therapeutic Molecular Biomarkers in Third Millennium: A Breakthrough in Gastric Cancer. *BioMed research international* 2017;2017:7869802. doi: 10.1155/2017/7869802 [published Online First: 2017/11/03]
9. Beyer K ML, Lardas M, Maclennan S, Monagas S, Antunes-Lopes T, et al. PIONEER's systematic review of outcomes reported in effectiveness trials for interventions in locally advanced prostate cancer. 11th European Multidisciplinary Congress on Urological Cancers. 11th European Multidisciplinary Congress on Urological Cancers. Vienna, 2019.
10. Beyer K MS, Lardas M, Moris L, Omar M, Flaherty S. PIONEER's update and integration of a localised prostate cancer core outcome set for effectiveness trials and a standard set for clinical practice. Annual Meeting of the European Association of Urology. Annual Meeting of the European Association of Urology. Barcelona2019, 2019.
11. Beyer K MS, Lardas M, Moris L, Vradi E, Picozzi M, et al. . PIONEER's update and integration of a metastatic Prostate Cancer Core Outcome Set. ICHOM 2019. Rotterdam, 2019.
12. Beyer K MS, Lardas M, Moris L, Omar M, MacLennan S, et al. PIONEER's update and integration of a localised prostate cancer core outcome set for effectiveness trials and a standard set for clinical practice. 11th European Multidisciplinary Congress on Urological Cancers. 11th European Multidisciplinary Congress on Urological Cancers. Vienna, 2019.
13. Beyer K MS, Lardas M, Moris L, Vradi E, Omar, I, et al PIONEER's systematic review of outcomes in RCTs of men with non-metastatic castration resistant prostate cancer. Is there a need for a core outcome set? Annual Meeting of the European Association of Urology. Amsterdam, 2020.
14. Epstein JI, Egevad L, Amin MB, et al. The 2014 International Society of Urological Pathology (ISUP) Consensus Conference on Gleason Grading of Prostatic Carcinoma: Definition of

For peer review only - http://bmjopen.bmj.com/site/about/guidelines.xhtml

Grading Patterns and Proposal for a New Grading System. *The American journal of surgical pathology* 2016;40(2):244-52. doi: 10.1097/pas.0000000000000530 [published Online First: 2015/10/23]
15. Moher D, Liberati A, Tetzlaff J, et al. Preferred reporting items for systematic reviews and meta-analyses: the PRISMA statement. *PLoS medicine* 2009;6(7):e1000097-e97. doi: 10.1371/journal.pmed.1000097
16. Higgins JPT TJ, Chandler J, Cumpston M, Li T, Page MJ, Welch VA Cochrane Handbook for Systematic Reviews of Interventions. Chichester (UK): Cochrane, 2019.
17. Moons KGM, de Groot JAH, Bouwmeester W, et al. Critical Appraisal and Data Extraction for Systematic Reviews of Prediction Modelling Studies: The CHARMS Checklist. *PLOS Medicine* 2014;11(10):e1001744. doi: 10.1371/journal.pmed.1001744
18. Riley RD, Moons KGM, Snell KIE, et al. A guide to systematic review and meta-analysis of prognostic factor studies. *BMJ (Clinical research ed)* 2019;364:k4597. doi: 10.1136/bmj.k4597 [published Online First: 2019/02/01]
19. Wolff RF, Moons KGM, Riley RD, et al. PROBAST: A Tool to Assess the Risk of Bias and Applicability of Prediction Model Studies. *Annals of Internal Medicine* 2019;170(1):51-58. doi: 10.7326/M18-1376
20. Moons KGM, Wolff RF, Riley RD, et al. PROBAST: A Tool to Assess Risk of Bias and Applicability of Prediction Model Studies: Explanation and Elaboration. *Ann Intern Med* 2019;170(1):W1-w33. doi: 10.7326/m18-1377 [published Online First: 2019/01/01]
21. Whiting PF, Rutjes AW, Westwood ME, et al. QUADAS-2: a revised tool for the quality assessment of diagnostic accuracy studies. *Ann Intern Med* 2011;155(8):529-36. doi: 10.7326/0003-4819-155-8-201110180-00009 [published Online First: 2011/10/19]
22. Hayden JA, van der Windt DA, Cartwright JL, et al. Assessing bias in studies of prognostic factors. *Ann Intern Med* 2013;158(4):280-6. doi: 10.7326/0003-4819-158-4-201302190-00009 [published Online First: 2013/02/20]
23. Peters MD, Godfrey CM, Khalil H, et al. Guidance for conducting systematic scoping reviews. *Int J Evid Based Healthc* 2015;13(3):141-6. doi: 10.1097/xeb.0000000000000050 [published Online First: 2015/07/03]
24. Arksey H, O'Malley L. Scoping studies: towards a methodological framework. *International Journal of Social Research Methodology* 2005;8(1):19-32. doi: 10.1080/1364557032000119616

For peer review only

For peer review only - http://bmjopen.bmj.com/site/about/guidelines.xhtml

Tables and Figures

Table 1: Search strategy

Database: OVID Medline Epub Ahead of Print, In-Process & Other Non-Indexed Citations, Ovid MEDLINE(R) Daily and Ovid MEDLINE(R) 1946 to Present, Embase <1974 to 2020 January 28>, EBM Reviews - Cochrane Database of Systematic Reviews <2005 to January 21, 2020>
Search Strategy:
----
1 exp *Prostatic Neoplasms/ (262435)
2 exp *prostate cancer/ (245472)
3 (prostat* adj2 (cancer* or carcinoma* or malignan* or tumor* or tumour* or neoplas* or adenocarcinoma* or adenoma*)).tw. (332251)
4 or/1-3 (366427)
5 ((diagnostic or prognos* or predict*) adj10 (biomarker or biomarkers or factor or factors)).tw,kw. (717487)
6 ((diagnostic or prognos* or predict*) adj10 (Oncotype Dx Prostate or Prolaris or Decipher or Decipher PORTOS or ProMark)).tw,kw. (458)
7 5 or 6 (717869)
8 4 and 7 (17456)
9 limit 8 to english language [Limit not valid in CDSR; records were retained] (16484)
10 limit 9 to yr="2014 -Current" (8417)
11 conference abstract.pt. or Congresses as Topic/ or Conference Review.pt. or "Journal: Conference Abstract".pt. (3815712)
12 10 not 11 (5902)
13 (exp animals/ or exp animal/ or exp nonhuman/ or exp animal experiment/ or animal model/ or animal tissue/ or non human/ or (rat or rats or mice or mouse or swine or porcine or murine or sheep or lambs or pigs or piglets or rabbit or rabbits or cat or cats or dog or dogs or cattle or bovine or monkey or monkeys or trout or marmoset$1).ti.) not (humans/ or human/ or human experiment/ or (human* or men or women or patients or subjects).tw.) (10251935)
14 12 not 13 (5882)
15 note/ or editorial/ or letter/ or Comment/ or news/ or (note or editorial or letter or Comment or news).pt. (4565255)
16 14 not 15 (5811)
17 (child/ or Pediatrics/ or Adolescent/ or Infant/ or adolescence/ or newborn/ or (baby or babies or child or children or pediatric* or paediatric* or peadiatric* or infant* or infancy or neonat* or

For peer review only - http://bmjopen.bmj.com/site/about/guidelines.xhtml

newborn* or new born* or adolescen* or preschool or pre-school or toddler*).tw.) not (adult/ or aged/ or (aged or adult* or elder* or senior* or men or women).tw.) (4146377)

18 16 not 17 (5794)

19 18 use ppez,oemezd (5788)

20 10 use coch (6)

21 19 or 20 (5794)

22 remove duplicates from 21 (3140)

Figure 1: Overview of four stage process

For peer review only

For peer review only - http://bmjopen.bmj.com/site/about/guidelines.xhtml

Figure 1: Overview of four stage process

Workflow	Task
Stage 1.	Broad literature-based systematic review of diagnostic and prognostic factors (DPFs) for all stages of prostate cancer from 2014 onwards (English only; humans).<br>• Extract data from the included studies following the CHARMS-PF guideline.
Stage 2.	Discussion of systematic review findings by a multidisciplinary expert panel<br>• Review the list of included studies
Stage 3.	Risk of Bias Assessment and applicability of individual studies using PROBAST, QUIPS and QUADAS-2
Stage 4.	Quantitative assessment of individual articles using meta-analytic techniques:<br>• If PROBAST indicates low risk of bias and low concerns for applicability:<br>Oxford Classification Centre for Evidence Based Medicine:<br>1. If there is Level 1a (SR of inception cohort studies validated in different populations or Level 1 diagnostic studies from different clinical centres), we do not do a meta-analysis<br>2. No Level 1a but >2 studies which apply to 1b (Prognostic: individual inception cohort study with > 80% follow-up; CDR" validated in a single population; Diagnostic: Validating cohort study with good reference standards; or CDR" tested within one clinical centre) or 1c (Prognostic: All or none case-series; diagnostic: Absolute SpPins and SnNouts), we do a meta-analysis
Final aim:	Develop online PIONEER Online Search Tool for DPFs

For peer review only

For peer review only - http://bmjopen.bmj.com/site/about/guidelines.xhtml

PRISMA-P (Preferred Reporting Items for Systematic review and Meta-Analysis Protocols) 2015 checklist: recommended items to address in a systematic review protocol*

Section and topic	Item No	Checklist item	Reported at section
**ADMINISTRATIVE INFORMATION**			**Page number**
Title:			
Identification	1a	Identify the report as a protocol of a systematic review	p.4
Update	1b	If the protocol is for an update of a previous systematic review, identify as such	n/a
Registration	2	If registered, provide the name of the registry (such as PROSPERO) and registration number	n/a
Authors:			
Contact	3a	Provide name, institutional affiliation, e-mail address of all protocol authors; provide physical mailing address of corresponding author	Title page- p.1
Contributions	3b	Describe contributions of protocol authors and identify the guarantor of the review	p.11
Amendments	4	If the protocol represents an amendment of a previously completed or published protocol, identify as such and list changes; otherwise, state plan for documenting important protocol amendments	n/a
Support:			
Sources	5a	Indicate sources of financial or other support for the review	p. 11
Sponsor	5b	Provide name for the review funder and/or sponsor	p. 11
Role of sponsor or funder	5c	Describe roles of funder(s), sponsor(s), and/or institution(s), if any, in developing the protocol	p. 11
**INTRODUCTION**			
Rationale	6	Describe the rationale for the review in the context of what is already known	p. 4
Objectives	7	Provide an explicit statement of the question(s) the review will address with reference to participants, interventions, comparators, and outcomes (PICO)	p. 4-5
**METHODS**			
Eligibility criteria	8	Specify the study characteristics (such as PICO, study design, setting, time frame) and report characteristics (such as years considered, language, publication status) to be used as criteria for eligibility for the review	p. 6-7
Information sources	9	Describe all intended information sources (such as electronic databases, contact with study authors, trial registers or other grey literature sources) with planned dates of coverage	p. 14
Search strategy	10	Present draft of search strategy to be used for at least one electronic database, including planned limits, such that it could be repeated	p. 14

For peer review only

Study records:			
Data management	11a	Describe the mechanism(s) that will be used to manage records and data throughout the review	p. 6
Selection process	11b	State the process that will be used for selecting studies (such as two independent reviewers) through each phase of the review (that is, screening, eligibility and inclusion in meta-analysis)	p.6
Data collection process	11c	Describe planned method of extracting data from reports (such as piloting forms, done independently, in duplicate), any processes for obtaining and confirming data from investigators	p.6
Data items	12	List and define all variables for which data will be sought (such as PICO items, funding sources), any pre-planned data assumptions and simplifications	p.6
Outcomes and prioritization	13	List and define all outcomes for which data will be sought, including prioritization of main and additional outcomes, with rationale	p.6-7
Risk of bias in individual studies	14	Describe anticipated methods for assessing risk of bias of individual studies, including whether this will be done at the outcome or study level, or both; state how this information will be used in data synthesis	p.8
Data synthesis	15a	Describe criteria under which study data will be quantitatively synthesised	p. 9
	15b	If data are appropriate for quantitative synthesis, describe planned summary measures, methods of handling data and methods of combining data from studies, including any planned exploration of consistency (such as I^2, Kendall's τ)	n/a
	15c	Describe any proposed additional analyses (such as sensitivity or subgroup analyses, meta-regression)	p. 9
	15d	If quantitative synthesis is not appropriate, describe the type of summary planned	p. 10
Meta-bias(es)	16	Specify any planned assessment of meta-bias(es) (such as publication bias across studies, selective reporting within studies)	n/a
Confidence in cumulative evidence	17	Describe how the strength of the body of evidence will be assessed (such as GRADE)	n/a

*** It is strongly recommended that this checklist be read in conjunction with the PRISMA-P Explanation and Elaboration (cite when available) for important clarification on the items. Amendments to a review protocol should be tracked and dated.** **The copyright for PRISMA-P (including checklist) is held by the PRISMA-P Group and is distributed under a Creative Commons Attribution Licence 4.0.**

From: Shamseer L, Moher D, Clarke M, Ghersi D, Liberati A, Petticrew M, Shekelle P, Stewart L, PRISMA-P Group. Preferred reporting items for systematic review and meta-analysis protocols (PRISMA-P) 2015: elaboration and explanation. BMJ. 2015 Jan 2;349(jan02 1):g7647.

For peer review only
